# Virtual and Augmented Reality in Social Skills Interventions for Individuals with Autism Spectrum Disorder: A Scoping Review

**DOI:** 10.1007/s10803-021-05338-5

**Published:** 2021-11-16

**Authors:** Anders Dechsling, Stian Orm, Tamara Kalandadze, Stefan Sütterlin, Roald A. Øien, Frederick Shic, Anders Nordahl-Hansen

**Affiliations:** 1 Faculty of Teacher Education and Languages, Østfold University College, B R A veien 4, 1757 Halden, Norway; 2 Department of Welfare and Participation, Western Norway University of Applied Sciences, Bergen, Norway; 3 Faculty of Computer Science, Albstadt-Sigmaringen University, Sigmaringen, Germany; 4 Faculty of Health, Welfare and Organisation, Østfold University College, Halden, Norway; 5 Department of Education, The Arctic University of Norway – University of Tromsø, Tromsö, Norway; 6 Child Study Center, Yale University School of Medicine, New Haven, USA; 7 Center for Child Health, Behavior and Development, Seattle Children’s Research Institute, Seattle, USA; 8 Department of Pediatrics, University of Washington School of Medicine, Washington, USA

**Keywords:** Autism spectrum disorder, Virtual reality, Augmented reality, Social skills

## Abstract

In the last decade, there has been an increase in publications on technology-based interventions for autism spectrum disorder (ASD). Virtual reality based assessments and intervention tools are promising and have shown to be acceptable amongst individuals with ASD. This scoping review reports on 49 studies utilizing virtual reality and augmented reality technology in social skills interventions for individuals with ASD. The included studies mostly targeted children and adolescents, but few targeted very young children or adults. Our findings show that the mode number of participants with ASD is low, and that female participants are underrepresented. Our review suggests that there is need for studies that apply virtual and augmented realty with more rigorous designs involving established and evidenced-based intervention strategies.

**Supplementary Information:**

The online version contains supplementary material available at 10.1007/s10803-021-05338-5.

## Introduction

Autism spectrum disorder (ASD; autism from hereon) refers to a spectrum of neurodevelopmental conditions marked by impairments in social communication and the presence of repetitive behavior patterns and interests (American Psychiatric Association, 2013). Individuals with autism are heterogeneous in their levels of general functioning. For example, some individuals with autism develop advanced expressive language ability, show more subtle difficulties in social interactions, and their repetitive behavior patterns are associated with adherence to routines in their daily life (Horwitz et al., 2020). Others may have impaired expressive language, extensive difficulties with social interaction, and their repetitive behavior takes the form of stereotyped movements (Hattier et al., 2011; Kjellmer et al., 2012).

Already at an early age, individuals with autism show tendencies of orienting themselves mostly to non-social stimuli, at the expense of social stimuli, and this orientation can have cascading effects on their social and linguistic development (Gale et al., 2019). The preference for non-social stimuli and restrictive behavior might be related to challenges for individuals with autism when encountering society. Individuals with autism commonly have lower quality peer friendships with fewer reciprocal relationships and lower acceptance by their classmates (Chamberlain et al., 2007). Difficulties individuals with autism experience in educational settings are often related to making sense of social stimuli and unpredictable social environments (Lüddeckens, 2020). In fact, individuals with autism show significantly more school refusal behavior than their neurotypical peers (Munkhaugen et al., 2017). A high prevalence of school refusal behavior can possibly be attributed to difficulties experienced at school, perhaps due to social difficulties in interaction with peers and teachers. Many individuals with autism experience bullying during their school years (Skafle et al., 2019).

The social communicative impairments defining the disorder makes social skills an important target in educational interventions (Wolstencroft et al., 2018). Many different definitions of social skills have emerged in the literature (Wolstencroft et al., 2018). The term social skill is used interchangeably with other terms, such as social competence and social functioning (see Cordier et al., 2015). In this review, we define the term social skills broadly as a behavior that is performed in a social context and involves interpersonal engagement (Cordier et al., 2015; Wolstencroft et al., 2018). This engagement is not restricted to live human-to-human interactions in this study since we also include human–computer interactions (e.g., engagement with robotic avatars etc.).

Technology, such as virtual reality (VR), seems promising in regard to practicing social skills (Howard & Gutworth, 2020), and represent cost effective ways of meeting the social and, ultimately, the educational needs of individuals with autism. VR technology displays artificial environments that may emulate real world scenarios by generating visual and auditory stimuli, with realistic images or other sensations that can surround the end user. Augmented reality (AR) provides artificial visual and auditory information superimposed on the veridical, real-world environment. AR is often presented through tablets and smartphones in addition to AR-glasses. VR can be presented with various tools such as Head-Mounted Displays (HMD) and Cave Automatic Virtual Environment (CAVE), and even desktop or laptop computers. VR HMDs are goggles presenting virtual environments that provide a sense of surrounding the user completely. CAVE is a tool that uses two-dimensional projected displayed arranged around the user to present a pseudo three-dimensional environment. VR/AR and CAVE all provide opportunities where the users can interact through specialized controllers, computer vision techniques (e.g., through motion capture techniques), and other computer-based sensing systems (e.g., eye-tracking, speech recognition etc.).

In the last decade, the number of publications including computer-based or VR/AR assessments and interventions involving individuals with autism has increased significantly (Dechsling et al., 2020). The majority of studies on autism and VR/AR focus on social and emotional skills (Lorenzo et al., 2018; Mesa-Gresa et al., 2018), with multiple studies reporting benefits through skill training (e.g., Didehbani et al., 2016; Kandalaft et al., 2013; Maskey et al., 2014; Newbutt et al., 2016; Yang et al., 2017). In addition, computer technology methods have been described as highly motivating for many individuals with autism (Dechsling et al., 2020; Newbutt et al., 2016, 2020; Yang et al., 2017).

A few previous reviews have summarized evidence on VR and assessments or interventions on social skills for individuals with autism. Miller and Bugnariu (2016) conducted a systematic literature search on autism and VR. The included 29 studies were published before July 2015 and focused on both assessments and interventions of social skills and graded them in different levels of the immersion on the apparatus used. The authors presented a useful table on classification of virtual environment characteristics by levels of immersion. An example of a low immersion virtual environment would be one delivered via computer monitor or tablet; a high immersion environment would be one presented through an HMD.

Mesa-Gresa and colleagues conducted a systematic review on the effectiveness of various types of VR-interventions for children with autism, including social skills (Mesa-Gresa et al., 2018), while Lorenzo et al. (2018) focused on identifying variables used in studies that applied VR for students with autism. After excluding studies with participants above 18 years of age in their review, Mesa-Gresa et al. (2018) included 31 studies and concluded that there is moderate evidence for the effectiveness of VR-interventions for children with autism. Lorenzo et al. (2018) on the other hand, included 12 studies and concluded that immersive virtual reality is a promising tool for improving the skills of students with autism. Nevertheless, they pointed out several weaknesses in the studies included, such as small sample sizes and control groups consisting of individuals without autism.

These reviews had some methodological issues limiting their results in terms of providing an exhaustive overview, such as the choice to exclude some age-cohorts. In addition, both reviews used a limited set of search databases. Mesa-Gresa et al. (2018) only searched three databases (Web of Science, PubMed and Scimago Journal & Country Rank), and not psychology and education specific databases such as PsycInfo and ERIC. Lorenzo et al. (2018) did not report which databases they searched. Based on the information they provided, it seems likely that only Web of Science was used with a search string limited to only two search terms.

The rapid development of the research field on autism and VR/AR (Dechsling et al., 2020) combined with the extensive number of journals publishing studies in this domain (both technology- and autism specialized as well as general journals), and the narrow search strategies in previous reviews, warrants a review to map and evaluate the extant research. A transparent review, targeting VR/AR interventions on social skills for individuals with autism, using more exhaustive search string and a more diverse set of search platforms than previous reviews. Scoping reviews are useful when navigating a complex and heterogeneous literature and are commonly conducted to summarize and explore the breadth and depth of the literature, identify gaps, and inform future research (Peters et al., 2020; Tricco et al., 2016).

The objectives of this review are to provide a comprehensive summary of studies where VR/AR-interventions have been used to improve social skills in individuals with autism; to evaluate the literature including the methodology used and the social skills targeted; and to identify research gaps in the literature.

## Method

### Search Strategy

An information retrieval specialist searched the databases: PsycINFO, PubMed, ERIC, Education Research Complete, IEEE Xplore, and the search platform Web of Science, on February 3rd, 2021. The following search string from Dechsling et al. (2021) was used: (Pervasive development disorder OR pdd OR pdd-nos OR pervasive developmental disorder not otherwise specified OR autism OR autistic OR autism spectrum disorder OR autism spectrum disorders OR Asperger OR asd OR autism spectrum condition* OR asc AND Virtual Reality OR vr OR hmd OR Head-mounted display OR Immersive Virtual Environment OR Augmented Reality OR Artificial Reality OR Oculus OR Immersive Technolog* OR Mixed Reality OR Hybrid Reality OR Immersive Virtual Reality System OR 3D Environment* OR htc vive OR cave OR Virtual Reality Exposure OR vre). The first and second author also searched the references in other reviews (Lorenzo et al., 2018; Mesa-Gresa et al., 2018; Miller & Bugnariu, 2016) for potential additional studies. No limitations regarding language or publication year were applied in the search, but the search was limited to peer-reviewed publications and to publications that included human participants.

### Inclusion and Exclusion of Studies

The study inclusion criteria when screening were as follows: (1) Peer-reviewed studies in English published 2010 or after, (2) included participants diagnosed with autism, (3) included interventions on social skills as defined below (4) used VR/AR-equipment (not restricted to HMD), and (5) involved a human–computer interaction to be evaluated as a VR/AR-intervention. The exclusion criteria were: (1) Grey literature (e.g., dissertations, posters, etc.), (2) studies published before 2010, (3) studies not written in English, (4) studies where the participants did not act or interact in the virtual environment, (4) studies where VR/AR skill improvement was not directly directed to individuals with autism (e.g. VR teaching of professionals working with various populations).

The inclusion criteria on social skills were widely defined as studies with the aim or outcome regarding some social interactions, such as communication, emotion recognition, joint attention, pretend play, and job interview skills. Feasibility studies, pilot studies, and peer-reviewed conference papers were included if they reported interventional data on participants.

### Screening and Data Extraction

The first and second author screened the publications from the search consulting the PRISMA guidelines (Moher et al., 2009). After removing duplicates, the initial search results were screened for titles and abstracts by the first and second author for relevance within ASD* and Virtual Reality*, and then selected studies were eventually screened for full text articles. Disagreements were resolved through discussions.

From the included studies, we extracted the following information: authors, publication year, study type (e.g., case-study, pilot-study, randomized controlled trials etc.), duration and aim of the intervention. Information about sample size, age range, and intelligence quotient (IQ) were also extracted, whenever applicable. In addition, we replicated the coding process from Dechsling et al. (2020) to assess the reported acceptability (“i.e., participant reports on usability, enjoyment, likeability, tolerability and so on”, p. 163) data in the included studies.

## Results

**Fig. 1:**
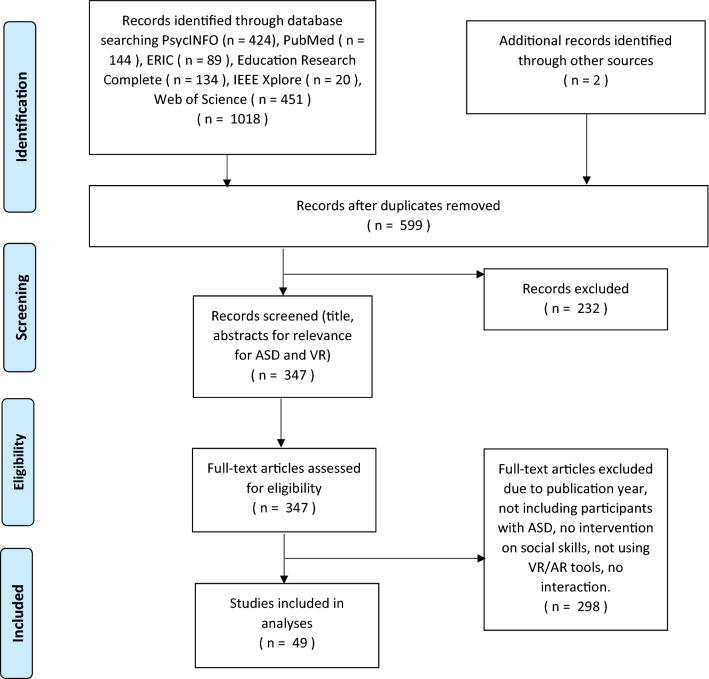
Flow chart of the selection process of included studies

After removing duplicates, the initial search resulted in 599 publications and the screening resulted in 49 publications meeting the inclusion criteria. See Flow chart (Fig. 1) for the details. One relevant article was included after manually searching the reference lists of included publications and the aforementioned reviews. The publications are published in psychology/psychiatry journals (19), educational journals (8), information technology journals (6), hybrid journals of education and technology (11), and the rest in miscellaneous journals (e.g., biomedicine, pediatrics, sociology). The matrix in the Supplementary Appendix contains an overview of the included articles.

The total number of participants with autism in the included publications was 652. The gender distribution of the participants in this sample were male n = 604, and female n = 48, meaning 7.4% of participants were female (which is lower than the reported male-to-female gender ratio of approximately 3:1 in autism [Loomes et al., 2017]). Note that the reported number of female participants in this total sample may be inaccurate due to 12% of the studies not reporting gender distribution.

**Fig. 2:**
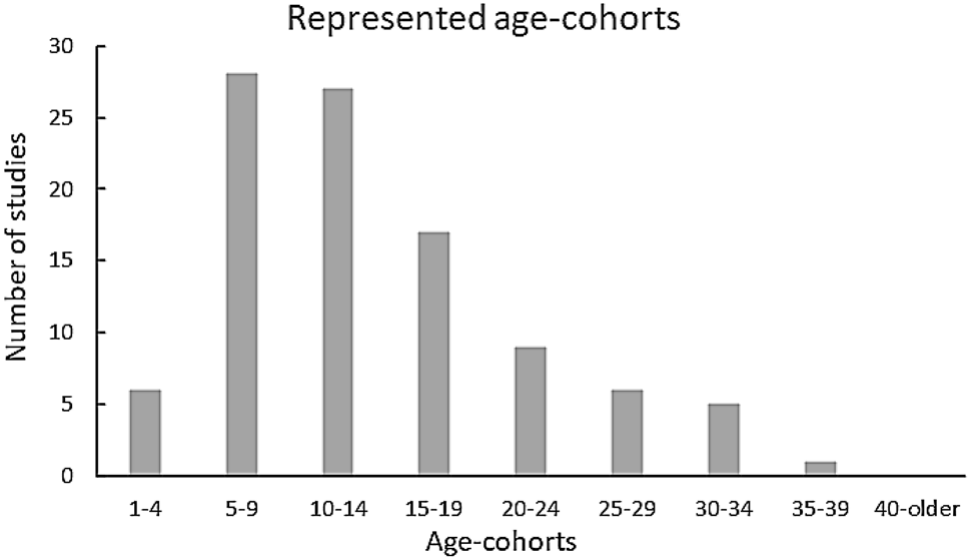
A bar chart of the number of studies and the respective represented age-cohorts. One to 4-year old participants appear in six studies, 5–9 and 10–14-year old participants appear in just over 25 studies, 15–19-year old participants appear in approximately 17 studies, 25–29- and 30–34-year old participants appear in approximately five studies each, and only one study include participants from 35 or older.

The mean and median number of participants with autism per publication were 13.3 and 10.0 respectively, range was 2–99 (note that if we exclude Burke et al. (2020) the range would be 2–40), and the mode number was 3 participants. The age of participants with autism ranged from 2 to 38 years old. The studies included mainly children and adolescents, with approximately 16% of the studies involving adult individuals with autism (20–38 years of age, Fig. 2).

The interventions were mostly directed towards individuals with intellectual functioning above the Intelligence Quotient (IQ) cut-off score for intellectual disability (IQ ≥ 70), and around the general population average. However, scarcely above half of the studies in the sample reported participants’ IQ-scores.

**Fig. 3:**
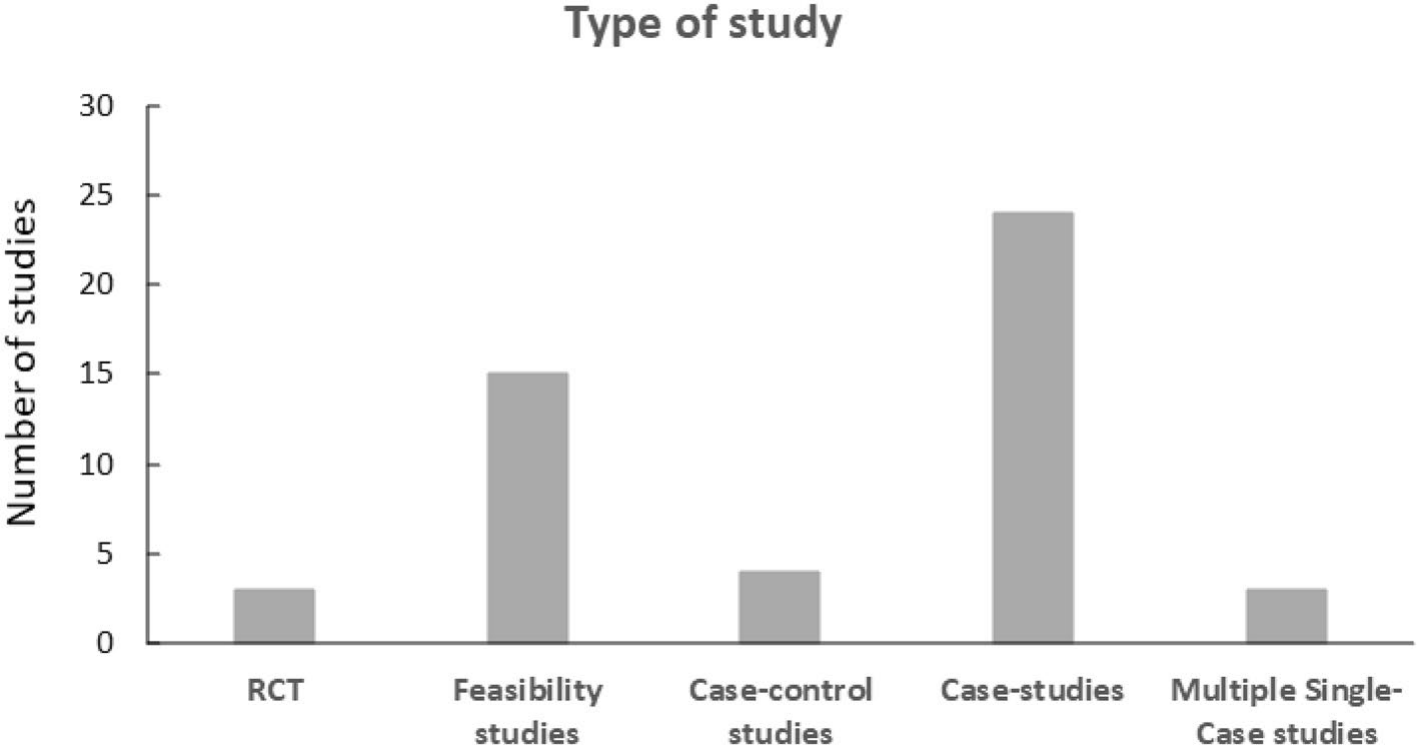
A bar chart over different types of study. Three RCT, fifteen feasibility studies, four case-control-studies, 24 case-studies, and three multiple single-case studies.

The majority of studies were case-studies and feasibility (pilot/exploratory) studies (Fig. 3), while only three randomized controlled trials (Smith et al., 2014; Strickland et al., 2013; White et al., 2016). Mean duration of the interventions was 8.75 weeks, ranging from < 1 to 40 weeks.

The studies in our sample targeted: (1) various social interaction skills (e.g., Ke & Moon, 2018; Lorenzo et al., 2019), joint attention (e.g., Amaral et al., 2018; Ravindran et al., 2019), job interview skills (e.g., Burke et al., 2018, 2020; Smith et al, 2014; Strickland et al., 2013; Ward & Esposito, 2019), pretend play (e.g., Bai et al., 2015), collaboration and communication (Bernardini et al., 2014; Crowell et al., 2019; Malinverni et al., 2017; Moon & Ke, 2019; Trepagnier et al., 2011; Zhao et al., 2018). Or (2) social knowledge such as emotion recognition (Chen et al., 2016; Didehbani et al., 2016; Serret et al., 2014), broader emotional competence (Lorenzo et al., 2016), and social cognition (Chen et al., 2016). Or (3) a combination of both such as emotion recognition and responses (Chen et al., 2015; Ip et al., 2018; Liu et al., 2017), social attention and gestures (Lee, 2020; Lee et al., 2018), or social understanding and cognition (Cheng et al., 2015; Kandalaft et al., 2013; Yang et al., 2017, 2018).

**Fig. 4:**
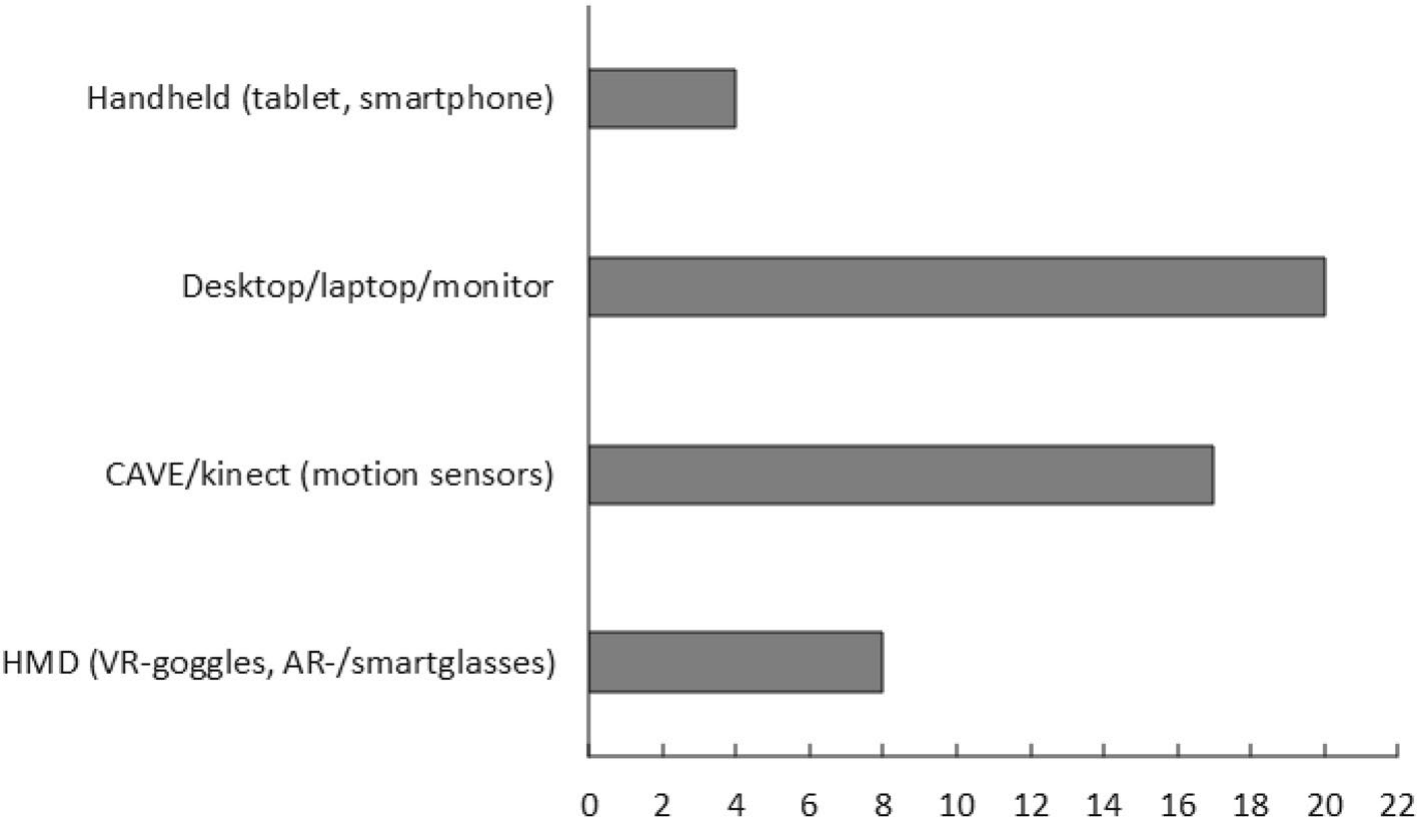
A bar chart of the different kinds of hardware used. Four studies using handheld equipment like tablets or smartphones. Twenty studies using desktops, laptops, or monitors. Seventeen studies using CAVE or Kinect (with motion sensors). Eight studies using Head-Mounted Displays such as VR-goggles or AR-goggles/smartglasses

The software and/or hardware that is used in the different studies are listed in Table 1 and reveal a wide range of equipment used aiming at providing a virtual reality environment or augmented reality (Fig. 4). Several levels of immersion were covered by this literature with low immersion apparatus and environments such as monitor-based interventions, and high-immersion apparatus such as VR in HMDs and CAVE. In between, one can find the use of motion cameras (Kinect), laptops with or without additional equipment such as joysticks, headsets and different kinds of software. Eight of the included studies used HMD’s, either AR-goggles, smart-glasses or VR-goggles.

**Table 1:** Included studies

Authors. (year), country of study origin	Study type/duration	Aim	Methodology	N* (f**)	Participant age range (mean IQ)	HMD/equipment used
Amaral et al. (2018), Portugal	Feasibility study/17 weeks	Joint attention	Joint attention through EEG brain computer interface	15 (0)	16–38 (102.53)	Yes/Oculus Rift + g.Nautilus EEG system
Babu and Lahiri (2020), India	Case–control study/80 min	Social interaction	Virtual reality multiplayer interaction platform with eye-gaze monitoring	18 (n/a)	n/a mean age 9.8 (n/a)	No/eye-tracker, multiplayer interaction framework
Bai et al. (2015), United Kingdom	Case-study (within subject)/n/a	Pretend play	Pretend play in an AR open-ended environment	12 (0)	4–7 (n/a)	No/AR monitor + logitech webcam + marker-based tracking
Beach and Wendt. (2016), USA	Case-study (qualitative)/4 weeks	Social interaction	Social skills through observing scenarios	2 (0)	15–18 (n/a)	Yes/Oculus Rift
Bernardini et al. (2014), United Kingdom	Feasibility study/6 weeks	Social communicative behaviors	Training social communicative behavior with virtual agents	19 (1)	4–14 (n/a)	No/Multitouch LCD display with eye-gaze tracking
Burke et al. (2018), USA	Case-study/14 weeks	Job interview skills	Job interview skills using Virtual Interactive Training Agents	22 (n/a)	19–31 (n/a)	No/Kinect + TV-screen
Burke et al. (2020), USA	Case-study (within subject)/22 weeks	Job interview skills	Job interview skills using Virtual Interactive Training Agents	99 (n/a)	n/a mean age 21.71 (n/a)	No/laptop, monitor with camera sensor
Chen et al. (2015), Taiwan	Case-study/6 weeks	Promote emotional expression and social skills	Promote emotional expressions through AR self-facial modeling	3 (0)	10–13 (100.67)	No/monitor + webcam
Chen et al. (2016), Taiwan	Case-study/7 sessions	Improve perception and judgement of facial expressions and emotions	Improve perceptions of non-verbal cues through AR video modeling	6 (0)	11–13 (103.66)	No/tablet
Cheng et al. (2015), Taiwan	Case-study (single subject)/6 weeks	Enhance social understanding and social skills	Enhance social skills using a 3D-social understanding	3 (0)	10–12 (82)	Yes/VR-goggles + laptop
Cheng et al. (2010), Taiwan	Case-study/23 weeks	Enhancing empathy (instructions)	Enhance empathy and social cognitions through collaborative and virtual environment	3 (0)	8–10 (104)	No/laptop computer
Cheng and Huang. (2012), Taiwan	Case-study/12 weeks	Joint attention	Joint attention through a virtual reality environment	3 (0)	9–12 (61.67)	No/Computer screen and data glove
Cheng and Ye. (2010), Taiwan	Feasibility (Pilot-study)/3 weeks	Social competence	Training social and emotional skills through a virtual learning environment	3 (0)	7–8 (102.33)	No/laptop computer
Chung et al. (2015), USA	Feasibility study (proof-of-concept)/5 weeks	Social behavior in cooperation in videogame	Peer cooperation in AR-videogame	3 (0)	8–12 (n/a)	No/AR game (Kinect)
Crowell et al. (2019), Spain	Case-study/120 min	Social skills, collaboration	Enhancing social skills in a virtual full-body interaction environment	25 (0)	4–14 (> 70)	No/Kinect
Didehbani et al. (2016), USA	Case-study/5 weeks	Emotion recognition and social attribution	Enhancing social skills through interactive VR-learning scenarios	30 (4)	7–16 (112.6)	No/Computer screen
Halabi et al. (2017), Qatar	Case-study/2 sessions of 20 min	Social interaction and communication	Enhancing communication skills through VR	3 (0)	4–6	Yes/CAVE VR + Oculus Rift
Herrero and Lorenzo (2020), Spain	Case–control study/10 sessions	Social and emotional abilities	Social interaction with avatars in a virtual environment	14 (1)	8–15 (approx. average)	Yes/Oculus Rift, smartphone
Ip et al. (2018), Hong Kong	Case-study/14 weeks	Enhance emotional and social adaption skills	Enhance social skills and emotion regulation in an immersive VR environment	36 (5)	6–12 (n/a)	No/4-side CAVE VR system
Kandalaft et al. (2013), USA	Feasibility study/5 weeks	Enhancing social skills, social cognition, and social functioning	Enhancing various social and social cognitive skills through VR-scenarios	8 (2)	18–26 (111.9)	No/Computer screen and VR software
Ke and Im. (2013), USA	Case-study/5 weeks	Social interaction	Social interaction tasks in VR-based learning environment	4 (2)	9–10 (n/a)	No/Computer screen and VR software
Ke and Lee. (2016), USA	Feasibility—exploratory case study/5 weeks	Social behavior in game play	Enhancing social skills through VR collaboration game	2 (0)	9–10 (n/a)	No/OpenSim
Ke and Moon. (2018), USA	Case-study/16 to 31 45–60 min sessions	Social interaction	Enhancing social skills through VR collaboration game	8 (1)	10–14 (> 70)	No/OpenSim
Ke et al. (2020), USA	Multiple single-case study/12 weeks	Social skills and role-play		7 (1)	10–14 (n/a)	No/OpenSim
Lee. (2020), Taiwan	Multiple single-case study/8 sessions	Judge other people’s social interaction and respond appropriately	Improving performance and understanding of body gestures through AR-role-play and tutoring	3 (1)	7–9 (102.3)	No/Kinect Tracking system
Lee et al. (2018), Taiwan	Case-study/6 weeks	Teach how to use social cues	Teaching social skills through social scenarios in AR	3 (1)	8–9 (93.3)	No/tablet, TV-screen and concept map
Liu et al. (2017), USA	Feasibility study/1 session	Emotion Recognition, Social Attention, Eye-contact and self-regulation	Enhancing social gaze and emotion recognition through AR-games	2 (0)	8–9 (n/a)	Yes/brain power system (BPS) + smartglasses
Lorenzo et al. (2019), Spain	Feasibility study (preliminary)/20 weeks	Social skills	Enhancing social skills through AR-based social activities	5 (1)	2–6 (n/a)	No/AR on smartphone
Lorenzo et al. (2016), Spain	Case–control/40 weeks	Emotional competence	Learning emotional scripts in VR	40 (11)	7–12 (n/a)	No/Semi-cave, robot and camera
Lorenzo et al. (2013), Spain	Case-study/40 weeks	Social competence	Training social and emotional skills in a VR-based school setting	20 (4)	8–15 (n/a)	No/3D IVE + Kinect + 3D glasses
Malinverni et al. (2017), Spain	Feasibility (Exploratory case study)/4 sessions	Social initiation	Enhancing social skills through VR collaborative game (PICO)	10 (0)	4–6 (95.9)	No/Pico's adventure Full body interact. Kinect
Milne et al. (2010), Australia	Case-study/n/a	Conversational skills and dealing with bullying	Conversational skills and dealing with bullying through virtual agent tutoring	14 (n/a)	6–15 (n/a)	No/desktop computer and webcam
Moon and Ke. (2019), USA	Feasibility (mixed methods)/6 sessions	Social skills; responding, initiating, negotiating and collaborating	Social skills training through social role-play in VR-game	15 (2)	10–14 (> 70)	No/open simulator
Parsons. (2015), United Kingdom	Case-study/2 weeks	Communicative perspective-taking skills	Social interaction in a collaborative VRE	6 (0)	10–13 (96)	No/laptop computers and microphone headsets
Ravindran et al. (2019), USA	Feasibility (pilot study)/5 weeks	Joint Attention	Training joint attention in VR	12 (0)	9–16 (n/a)	Yes/HMD cardboard with smartphone, tablets
Serret et al. (2014), France	Feasibility study (pilot-study)/4 weeks	Emotion recognition	Training emotion recognition through Serious game	33 (2)	6–17 (70.5)	No/computer game with a gamepad
Smith et al. (2014), USA	Randomized control trial/10 h	Job interview skills	Computer simulation of job interviews in VR	26 (n/a)	18–31 (n/a)	No/computer
Stichter et al. (2014), USA	Case-study/17 weeks	Social competence	Training social and emotional skills through distance education in iSocial	11 (0)	11–14 (99.55)	No/computers with headset
Strickland et al. (2013), USA	Randomized study/n/a	Job interview skills	Computer simulation of job interviews in VR	11 (0)	16–19 (n/a)	No/computer
Trepagnier et al. (2011), USA	Feasibility study/2 weeks	Conversational skills	Training of conversational skills through computer simulation	16 (1)	16–30 (109.4)	No/computer screen simulation
Tsai et al. (2020), Taiwan	Multiple single-case design/5 weeks	Emotion recognition	Role-play and observation	3 (0)	7–9 (87)	No/CAVE-like environment using Kinect
Uzuegbunam et al. (2018), USA	Feasibility (Pilot-study)/7 weeks	Social greeting behavior	Social greeting behavior through social narratives	3 (0)	7–11 (n/a)	No/touchscreen computer with Kinect v2
Vahabzadeh et al. (2018), USA	Feasibility study/6 weeks	Irritability, hyperactivity, and social withdrawal	Training on facial attention, mutual gaze and emotion recognition through smartglasses with social cues	4 (0)	6–8 (n/a)	Yes/smartglasses
Wang et al. (2017), USA	Case-study/18 weeks	Verbal and nonverbal social interaction	Training social and emotional skills through iSocial	11 (0)	11–14 (> 75)	No/3D VLE, computer screen
Ward and Esposito. (2019), USA	Exploratory study—case study/mean 129 min	Job interview skills	Computer simulation of job interviews in VR	12 (2)	18–22 (95.6)	No/Chromebook, headset w/microphone
White et al. (2016), USA	Randomized control trial/10–14 weeks	Social competence and self-regulation	Training of social interaction and emotion recognition in VE	8 (3)	19–23 (126.75)	No/desktop computer, tablet and EEG
Yang et al. (2017, 2018), USA	Case-study/5 weeks	Social cognition	Training social and emotional skills in immersive role play	17 (2)	18–31 (109.65)	No/computer and fMRI
Zhang et al. (2018a, 2018b), USA	Case–control study—Feasibility study/50 min	Collaborative interaction and verbal communication	Social communication through collaborative playing in a virtual environment	7 (1)	7–17 (191.06)	No/Desktop computers
Zhao et al. (2018), USA	Case-dyad study—feasibility study/n/a	Communication and collaboration	Collaborative skills through game-playing in VR	12 (n/a)	n/a mean age 12.20 (n/a)	No/motion controller, eye tracking, headset, webcam and computer

Table of the included studies

*Total number of participants with ASD (all genders) in the study, this number is excluding the other participants in the respective studies

**Number of female participants. This number is also included in the total number and should not be added to the total number of participants

Acceptability was reported in 21 of the 49 included studies. None of the studies reported negative evaluations, while 81% of the studies reported positive evaluations, and 19% reported inconclusive evaluations.

## Discussion

The aim of this scoping review was to identify intervention studies using VR/AR to improve social skills for individuals with autism. We identified 49 studies with various focus, intervention types, and technology within the scope of autism and social skills. Although this field of research is young, the median and mode number of participants across studies are noteworthy as the number of participants in such quantitative studies could be quite essential as a prerequisite for reliability and validity. Apart from eight studies like for example Burke et al. (2020), Ip et al. (2018), and Lorenzo et al. (2016), most of the studies are characterized with a small number of participants and short duration of the interventions.

Another remarkable finding is that the female participants with autism are underrepresented in our sample considering the male-to-female ratio within the autism spectrum. There might be several reasons why female participants are underrepresented. Some suggest females with autism to have less overt challenges related to social interaction, or that there are differences in social strategies between genders which makes the challenges more difficult to detect in females (Hiller et al., 2016), and that such strategies might camouflage other symptoms (Rynkiewicz et al., 2016). Therefore, it could be issues related to the likelihood of females being discovered in a selection process. However, May et al. (2016) found no differences in social ability between genders with autism, but some differences in communication ability, so there seems to be no reason to exclude females from this field of research based on differences in symptoms between genders. For example, Kanfiszer et al. (2017) interviewed several women with autism that explained challenges in daily life due to lack of skills and confidence when interacting with others. It is of course important to remember that such challenges should be met with both enhancing the individual’s skills, and with an increased awareness by the society. We have pointed to the novelty and rapid growth of the research field on social skills intervention in VR/AR, and as the evidence syntheses are emerging, there is a risk of unnoticed gender bias being part of such intervention practices. The underrepresentation of females should be seen as one shortcoming within this research area. Future research should have a greater emphasis and focus on recruiting female participants to balance the gender representation according to the gender ratio. It also calls for an investigation of which factors are present during the recruitment and selection process of participants, and furthermore investigations on elements important for females using VR/AR.

Further, this review shows that very few studies include individuals scoring below the threshold for an Intellectual Disability (ID) diagnosis. A selection bias of participants above this threshold for ID is found in most areas of autism research (Russell et al., 2019), and it seems that social skills interventions using VR/AR are no exception. Our findings could therefore be argued to complement Russell et al.’ (2019) and add this research area of technological interventions to the list of selection biased autism research.

Most of the studies focus on training various skills for social interaction. Play skills and job-related social skills are subdomains with few VR-intervention studies while job-interview skills are more frequent. There might be several factors that could influence the difficulties in implementing and intervening play skill-training into the computerized virtual environment. For instance, play skills are traditionally most relevant for younger children, which are least likely to tolerate the equipment, and understand and act upon the virtual environment. Interventions on job-related social skills are on the other hand an up-and-coming area of research (Grob et al., 2019). Emotional skills however, a domain that has been popular in computer-based interventions for individuals with autism (Ramdoss et al., 2012), are targeted by fewer studies included in this review. This could perhaps be because the development of VR/AR interventions have focused on skills where the traditional computer-based interventions fall short.

It is difficult to compare studies on social skills due to inconsistent definitions and intervention strategies (Ke et al., 2018). Technology-based interventions on social skills are no exception given the number of feasibility-, usability-, and pilot-studies in our overall sample, which confirms that the research field is still youthful. The novelty of the field can also explain the relatively low number of participants in most of the studies. There is a need for studies with more participants, which also use participants with autism as controls as opposed to using participants without autism. Only one study (Amaral et al., 2018) included participants in the age cohorts above 31 years old which indicates that the research field of using VR/AR on social skills with adults with autism over 30 is an untouched research area.

A variety of methods should be used when establishing causation since no method can reveal all relevant information (Anjum et al., 2020). Variation in types of studies should be considered a strength in evidence-based approaches. Since the majority of studies in our review are case- or feasibility studies, there is a need for more randomized controlled trials, which take into account necessary quality considerations (see for example Sandbank et al., 2020), to conclude on the effectiveness of using VR/AR. However, more replication studies, and studies using statistical methods developed for trials using rigorous single-case and multiple single-case designs (e.g., Shadish et al., 2014) can also contribute to building the evidence base in this research area. We welcome a diversity of studies which will impact the rigour and the generalizability of research applying VR/AR for individuals with autism.

The recency of the field can also explain the variety of methodology applied in the different studies in our sample. A variety of methodology is not a problem in itself, rather a strength once there is a sufficient number of studies converging towards the same outcome. However, an approach on using VR/AR in already established and evidence-based interventions might be warranted. For instance, Sandbank et al. (2020) concluded in their meta-analysis that there is a need for connecting technological tools with established intervention approaches and theoretical groundwork. Dechsling et al. (2021) provide examples on how to apply VR in Naturalistic Developmental Behavioral Interventions (NDBI; see Schreibman et al., 2015), the intervention approach considered most promising for helping children with autism in regard to social communication (Sandbank et al., 2020). Researchers and interventionists using VR/AR-technology should look at the most promising intervention approaches available, such as NDBI, when developing their intervention protocols and hereunder software development, with appropriate cultural adjustments. To obtain this there might be a need for clinical researchers and technology researchers to collaborate across disciplines in developing and designing robust VR/AR intervention studies. But most importantly, researchers should focus on involving individuals with autism in decision-making and development of such studies and technology (Parsons et al., 2020). In addition, strategies on assessing acceptability and stakeholder’s opinions should be implemented.

After summarizing the reports of participants with autism, across 63 studies utilizing VR/AR/computer-based tools in miscellaneous ways, Dechsling et al. (2020) found that computer-based equipment and HMDs were feasible and widely accepted amongst individuals with autism. Those findings are in accordance with the reports from the sample in our review showing a high acceptance rate among the studies reporting acceptability. However, an important note is that only 43% of the studies report such data. Recent findings from Newbutt et al. (2020) suggest further that individuals with autism prefer HMDs with controllers rather than tablets and HMDs with less opportunities to interact. However, HMDs such as VR/AR-goggles are only used in 16% of the studies in our overall sample. This indicates a need for more research on the effectiveness using high-immersive technology, in line with the conclusions of Miller and Bugnariu (2016). AR-/smartglasses are used in very few studies and research should investigate for example whether such glasses could be beneficial in aiding individuals with social cues in daily social encounters. Further considerations regarding cost and benefits using high-immersive versus low-immersive technological tools both in research and clinical practice should also be done, as well as other normative considerations (Dechsling et al., 2020). Individual considerations should be taken into account when applying various technologies. Some individuals may for example have sensory issues that makes a high immersive CAVE more feasible than HMDs (Dechsling et al., 2021).

The number of studies found both in computer-related journals and autism related journals shows the need for a broad search in databases and journals when reviewing studies on VR/AR, and autism. Our review identified a higher number of studies than the previous reviews, indicating a rapid growth of research in this area. This growth can be illustrated by the fact that the studies published from 2018 represent 41% of the total studies included in our review. The rapid growth of research in VR/AR is probably due to technological advancements making VR/AR software and equipment (e.g., HMDs) more affordable and widely available (Dechsling et al., 2020; Howard & Gutworth, 2020). These technological advancements offer new possibilities, for example can researchers and clinicians create an indefinite amount of different real-world scenarios VR intervention programs, in many cases also scenarios impossible to incorporate into traditional interventions (e.g., accident simulation, flights etc.; Manca et al., 2013). In regards to autism, an increased awareness of the possibilities combined with an awareness of the acceptability from individuals with autism, hereunder better equipment reducing notions of cybersickness, have contributed to the increased use of VR/AR in autism research. In social skills training in particular, VR/AR represents new possibilities for the development of social skills training programs where clients easily can get extensive and intensive training on social skills in situations and environments that mimics real-life. As pointed out by Dechsling et al. (2021), new VR/AR interventions can be based on existing evidence-based practices, and thus make them more affordable and easier to provide, broader availability at remote locations, and at the same time reduce unwanted geographical variation between clinics and service sites. However, as noted by the informants in Parsons et al. (2020), future research and development should focus on participatory design and to include stakeholders which also communicate without using speech since it is important also to include the opinions of marginalized groups.

## Limitations

The main limitations of this review are (1) omitting the grey literature search, and (2) restricting the field on publication year. Excluding grey literature might have biased our sample of included studies because, traditionally, studies with significant and novel results have greater chance to be published (i.e., “file drawer problem”; Rosenthal, 1979). Similarly, we could have excluded some relevant studies that have been published before 2010, which might have weakened the comprehensiveness of this study. Also, we did not conduct a meta-analysis on effects of interventions. However, considering the scarcity of rigorous designs, most notably the low amount of RCTs and lack of comparison groups with individuals with autism (Nordahl-Hansen et al., 2020), conducting a meta-analysis of intervention effects is still premature.

## Conclusion

This scoping review reveals that the number of studies using VR/AR in social skills intervention for individuals with autism have continued to increase, but that there are still gaps to be addressed (see Table 2 for a summary of the identified gaps). We have identified work by several authors and research teams, and a heterogeneity in terms of demographics, study aims and methodology. Still, a minority of the studies apply HMDs, but we suggest future studies investigate these tools further as soft- and hardware developments have increased the usability of HMDs.

**Table 2:** Identified gaps in the reviewed literature

Identified gap	Reason	Report	Percentage of studies reporting	Comment
Acceptability data	Too few reporting data	4/5 evaluate as positive	< 50%	Should be reported in every study
Diversity of methodology and demographics				
Female participants	Too few female participants	7.4% females	> 85%	The gender ratio in the total sample does not reflect the actual male-to-female ratio in autism
Studies with participants with ID	Few studies	One study report to have participants with ID	< 55%	May be underreported and underrepresented
Age above 30	Few studies	Six studies with participants above 30	100%	Lack of diversity in terms of participants’ age
HMD	Few studies	Eight studies applying VR-goggles or AR-/smartglasses	100%	Considering the reported acceptability and preference towards HMD
Diversity of methods				
RCT and case–control studies	Few studies	Seven RCTs or case–control studies	100%	Low amount compared to feasibility and case-studies (n = 39)
Multiple single-case design	Few studies	Three studies	100%	Low amount compared to feasibility and case-studies (n = 39)

The table summarized the identified gaps in the research literature on autism, VR/AR and social skills

It is important to consider the relatively low representation of female participants, and efforts should be made to recruit more gender-balanced samples. Furthermore, researchers and practitioners should strive towards identifying potential gender differences that might influence the effects. There is a need to investigate further the effectiveness of using VR/AR with individuals below ID threshold as they are also underrepresented in this research area as well.

Most studies lack a clear theoretical grounding in evidence-based approaches and we suggest more studies using rigorous designs, and studies including more participants with individuals with autism as controls. More interventions are needed for different age groups, most notably younger children and older adults. As the acceptance and feasibility of using VR/AR are heavily documented, it is time to utilize this technology in established evidence-based practices aiming at enhancing social skills helping individuals with autism maneuver in society. We recommend that future research build on collaborative interdisciplinary efforts including technology and software developers, autism researchers and individuals with autism.

## Supplementary Information

Below is the link to the electronic supplementary material.

Supplementary file 1 (DOCX 31 kb)
