# Supplementary material for: Virtual and Augmented Reality in Social Skills Interventions for Individuals with Autism Spectrum Disorder: A Scoping Review
Source: J Autism Dev Disord. 2021 Nov 16;52(11):4692–707. doi: 10.1007/s10803-021-05338-5 (PMC9556391; doi:10.1007/s10803-021-05338-5)
Supplement: Supplementary file 1 — (DOCX 31 kb) [file 10803_2021_5338_MOESM1_ESM.docx]

| *Appendix* | | | | |
| --- | --- | --- | --- | --- |
| **Authors.** | **Year** | **Title** | **Journal** | **Doi/URL** |
| Amaral et al. | 2018 | A Feasibility Clinical Trial to Improve Social Attention in Autistic Spectrum Disorder (ASD) Using a Brain Computer Interface. | Frontiers in Neuroscience | https://dx.doi.org/10.3389%2Ffnins.2018.00477 |
| Babu & Lahiri | 2020 | Multiplayer Interaction Platform With Gaze Tracking for Individuals With Autism. | IEEE transactions on neural systems and rehabilitation engineering : a publication of the IEEE Engineering in Medicine and Biology Society | https://doi.org/10.1109/TNSRE.2020.3026655 |
| Bai et al. | 2015 | Using Augmented Reality to Elicit Pretend Play for Children with Autism. | Ieee Transactions on Visualization and Computer Graphics, | https://doi.org/10.1109/tvcg.2014.2385092 |
| Beach and Wendt | 2016 | Using Virtual Reality to Help Students with Social Interaction Skills. | Journal of the International Association of Special Education | https://eric.ed.gov/?id=EJ1153717 |
| Bernadini et al. | 2014 | ECHOES: An intelligent serious game for fostering social communication in children with autism. | Information Sciences | https://doi.org/10.1016/j.ins.2013.10.027 |
| Burke et al. | 2018 | Using Virtual Interactive Training Agents (ViTA) with Adults with Autism and Other Developmental Disabilities. | Journal of Autism and Developmental Disorders | https://doi.org/10.1007/s10803-017-3374-z |
| Burke et al. | 2021 | Brief Report: Improving Employment Interview Self-efficacy Among Adults with Autism and Other Developmental Disabilities Using Virtual Interactive Training Agents (ViTA). | Journal of Autism and Developmental Disorders | https://doi.org/10.1007/s10803-020-04571-8 |
| Chen et al. | 2015 | Augmented reality-based self-facial modeling to promote the emotional expression and social skills of adolescents with autism spectrum disorders. | Research in Developmental Disabilites | https://doi.org/10.1016/j.ridd.2014.10.015 |
| Chen et al. | 2016 | Augmented reality-based video-modeling storybook of nonverbal facial cues for children with autism spectrum disorder to improve their perceptions and judgments of facial expressions and emotions. | Computers in Human Behavior | https://doi.org/10.1016/j.chb.2015.09.033 |
| Cheng et al. | 2015 | Using a 3D immersive virtual environment system to enhance social understanding and social skills for children with autism spectrum disorders. | Focus on Autism and Other Developmental Disabilities | https://doi.org/10.1177%2F1088357615583473 |
| Cheng et al. | 2010 | Enhancing empathy instruction using a collaborative virtual learning environment for children with autistic spectrum conditions. | Computers & Education | https://doi.org/10.1016/j.compedu.2010.06.008 |
| Cheng and Huang | 2012 | Using virtual reality environment to improve joint attention associated with pervasive developmental disorder. | Research in Developmental Disabilities | https://doi.org/10.1016/j.ridd.2012.05.023 |
| Cheng and Ye | 2010 | Exploring the social competence of students with autism spectrum conditions in a collaborative virtual learning environment - The pilot study. | Computers & Education | https://doi.org/10.1016/j.compedu.2009.10.011 |
| Chung et al. | 2015 | Social behaviors and active videogame play in children with autism spectrum disorder. | Games for Health | https://doi.org/10.1089/g4h.2014.0125 |
| Crowell et al. | 2019 | Structuring collaboration: Multi-user full-body interaction environments for children with Autism Spectrum Disorder. | Research in Autism Spectrum Disorders | https://doi.org/10.1016/j.rasd.2018.11.003 |
| Didehbani et al. | 2016 | Virtual Reality Social Cognition Training for children with high functioning autism. | Computers in Human Behavior | https://doi.org/10.1016/j.chb.2016.04.033 |
| Halabi et al. | 2017 | Design of Immersive Virtual Reality System to Improve Communication Skills in Individuals with Autism. | International Journal of Emerging Technologies in Learning | https://online-journals.org/index.php/i-jet/article/view/6766 |
| Herrero and Lorenzo | 2020 | An immersive virtual reality educational intervention on people with autism spectrum disorders (ASD) for the development of communication skills and problem solving. | Education and Information Technologies | https://doi.org/10.1007/s10639-019-10050-0 |
| Ip et al. | 2018 | Enhance emotional and social adaptation skills for children with autism spectrum disorder: A virtual reality enabled approach. | Computers & Education | https://doi.org/10.1016/j.compedu.2017.09.010 |
| Kandalaft et al. | 2013 | **Virtual Reality Social Cognition Training for young adults with high-functioning autism.** | **Journal of Autism and Developmental Disorders** | [10.1007/s10803-012-1544-6](https://doi.org/10.1007/s10803-012-1544-6) |
| Ke and Im | 2013 | Virtual-reality-based social interaction training for children with high-functioning autism. | The Journal of Educational Research | https://doi.org/10.1177%2F0162643420945603 |
| Ke and Lee | 2016 | Virtual reality based collaborative design by children with high-functioning autism: Design-based flexibility, identity, and norm construction. | Interactive Learning Environments | https://doi.org/10.1080/10494820.2015.1040421 |
| Ke and Moon | 2018 | Virtual Collaborative Gaming as Social Skills Training for High-Functioning Autistic Children. | British Journal of Educational Technology | https://doi.org/10.1111/bjet.12626 |
| Ke et al. | 2020 | Virtual Reality-Based Social Skills Training for Children With Autism Spectrum Disorder. | Journal of Special Education Technology | https://doi.org/10.1177/0162643420945603 |
| Lee | 2020 | Kinect-for-windows with augmented reality in an interactive roleplay system for children with an autism spectrum disorder. | Interactive Learning Environments | https://doi.org/10.1080/10494820.2019.1710851 |
| Lee et al. | 2018 | Augmented Reality Plus Concept Map Technique to Teach Children with ASD to Use Social Cues When Meeting and Greeting. | Asia-Pacific Education Researcher | https://doi.org/10.1007/s40299-018-0382-5 |
| Liu et al. | 2017 | Feasibility of an Autism-Focused Augmented Reality Smartglasses System for Social communication and Behavioral coaching. | Frontiers in Pediatrics | https://doi.org/10.3389/fped.2017.00145 |
| Lorenzo et al. | 2019 | Preliminary Study of Augmented Reality as an Instrument for Improvement of Social Skills in Children with Autism Spectrum Disorder. | Education and Information Technologies | https://doi.org/10.1007/s10639-018-9768-5 |
| Lorenzo et al. | 2016 | Design and application of an immersive virtual reality system to enhance emotional skills for children with autism spectrum disorders. | Computers & Education | https://doi.org/10.1016/j.compedu.2016.03.018 |
| Lorenzo et al. | 2013 | Inclusion of immersive virtual learning environments and visual control systems to support the learning of students with Asperger syndrome. | Computers & Education | https://doi.org/10.1016/j.compedu.2012.10.028 |
| Malinverni et al. | 2017 | An inclusive design approach for developing video games for children with Autism Spectrum Disorder. | Computers in Human Behavior | https://doi.org/10.1016/j.chb.2016.01.018 |
| Milne et al. | 2010 | Development of a virtual agent based social tutor for children with autism spectrum disorders. | The 2010 International Joint Conference on Neural Networks (IJCNN) | https://doi.org/10.1109/IJCNN.2010.5596584 |
| Moon and Ke | 2019 | Exploring the treatment integrity of virtual reality-based social skills training for children with high-functioning autism. | Interactive Learning Environments | https://doi.org/10.1080/10494820.2019.1613665 |
| Parsons | 2015 | Learning to work together: Designing a multi-user virtual reality game for social collaboration and perspective-taking for children with autism. | International Journal of Child-Computer Interaction | https://doi.org/10.1016/j.ijcci.2015.12.002 |
| Ravindran et al. | 2019 | Virtual Reality Support for Joint Attention Using the Floreo Joint Attention Module: Usability and Feasibility Pilot Study. | JMIR Pediatr Parent | https://doi.org/10.2196/14429 |
| Serret et al. | 2014 | Facing the challenge of teaching emotions to individuals with low- and high-functioning autism using a new Serious game: a pilot study. | Molecular Autism | https://doi.org/10.1186/2040-2392-5-37 |
| Smith et al. | 2014 | Virtual Reality Job Interview Training in Adults with Autism Spectrum Disorder. | Journal of Autism & Developmental Disorders | https://doi.org/10.1007/s10803-014-2113-y |
| Stichter et al. | 2014 | iSocial: Delivering the Social Competence Intervention for Adolescents (SCI-A) in a 3D Virtual Learning Environment for Youth with High Functioning Autism. | Journal of Autism & Developmental Disorders | https://doi.org/10.1007/s10803-013-1881-0 |
| Strickland et al. | 2013 | JobTIPS: A Transition to Employment Program for Individuals with Autism Spectrum Disorders. | Journal of Autism & Developmental Disorders | https://doi.org/10.1007/s10803-013-1800-4 |
| Trepagnier et al. | 2011 | Virtual Conversation Partner for Adults with Autism. | Cyberpsychology, Behavior, and Social Networking | <https://doi.org/10.1089/cyber.2009.0255> |
| Tsai et al. | 2020 | Inclusion of third-person perspective in CAVE-like immersive 3D virtual reality role-playing games for social reciprocity training of children with an autism spectrum disorder. | Universal Access in the Information Society | https://doi.org/10.1007/s10209-020-00724-9 |
| Uzuegbunam et al. | 2018 | MEBook: Multimedia Social Greetings Intervention for Children with Autism Spectrum Disorders. | Ieee Transactions on Learning Technologies | https://doi.org/10.1109/TLT.2017.2772255 |
| Vahabzadeh et al. | 2018 | Improved Socio-Emotional and Behavioral Functioning in Students with Autism Following School-Based Smartglasses Intervention: Multi-Stage Feasibility and Controlled Efficacy Study. | Behavioral Sciences | https://doi.org/10.3390/bs8100085 |
| Wang et al. | 2017 | Fostering verbal and non-verbal social interactions in a 3D collaborative virtual learning environment: a case study of youth with Autism Spectrum Disorders learning social competence in iSocial. | Educational Technology Research and Development | https://doi.org/10.1007/s11423-017-9512-7 |
| Ward and Esposito | 2019 | Virtual Reality in Transition Program for Adults with Autism: Self-Efficacy, Confidence, and Interview Skills. | Contemporary School Psychology | https://doi.org/10.1007/s40688-018-0195-9 |
| White et al. | 2016 | Psychosocial and computer-assisted intervention for college students with autism spectrum disorder: Preliminary support for feasibility. | Education and Training in Autism and Developmental Disabilities | http://www.ncbi.nlm.nih.gov/pmc/articles/pmc5241080/ |
| Yang et al. | 2017 | Brain responses to biological motion predict treatment outcome in young adults with autism receiving Virtual Reality Social Cognition Training: Preliminary findings. | Behaviour Research and Therapy | https://doi.org/10.1016/j.brat.2017.03.014 |
| Yang et al. | 2018 | Neural mechanisms of behavioral change in young adults with high-functioning autism receiving virtual reality social cognition training: A pilot study. | Autism Research | https://doi.org/10.1002/aur.1941 |
| Zhang et al. | 2018a | Design and Evaluation of a Collaborative Virtual Environment (CoMove) for Autism Spectrum Disorder Intervention. | Acm Transactions on Accessible Computing | https://doi.org/10.1145/3209687 |
| Zhang et al. | 2018b | Understanding Performance and Verbal-Communication of Children with ASD in a Collaborative Virtual Environment. | Journal of Autism & Developmental Disorders | https://doi.org/10.1007/s10803-018-3544-7 |
| Zhao et al. | 2018 | Hand-in-Hand: A Communication-Enhancement Collaborative Virtual Reality System for Promoting Social Interaction in Children With Autism Spectrum Disorders. | Ieee Transactions on Human-Machine Systems | https://doi.org/10.1109/thms.2018.2791562 |
